# The Effect of Prenatal and Childhood Development on Hearing, Vision and Cognition in Adulthood

**DOI:** 10.1371/journal.pone.0136590

**Published:** 2015-08-24

**Authors:** Piers Dawes, Karen J. Cruickshanks, David R. Moore, Heather Fortnum, Mark Edmondson-Jones, Abby McCormack, Kevin J. Munro

**Affiliations:** 1 School of Psychological Sciences, University of Manchester, Manchester, United Kingdom; 2 Population Health Sciences and Ophthalmology and Visual Sciences, School of Medicine and Public Health, University of Wisconsin, Madison, Wisconsin, United States of America; 3 Cincinnati Children’s Hospital Medical Center and Department of Otolaryngology, University of Cincinnati College of Medicine, Cincinnati, Ohio, United States of America; 4 NIHR Nottingham Hearing Biomedical Research Unit, Nottingham, United Kingdom; 5 MRC Institute of Hearing Research, Nottingham, United Kingdom; 6 Otology and Hearing Group, Division of Clinical Neuroscience, School of Medicine, University of Nottingham, Nottingham, United Kingdom; 7 Central Manchester Universities Hospitals NHS Foundation Trust, Manchester, United Kingdom; Leibniz Institute for Neurobiology, GERMANY

## Abstract

It is unclear what the contribution of prenatal versus childhood development is for adult cognitive and sensory function and age-related decline in function. We examined hearing, vision and cognitive function in adulthood according to self-reported birth weight (an index of prenatal development) and adult height (an index of early childhood development). Subsets (N = 37,505 to 433,390) of the UK Biobank resource were analysed according to visual and hearing acuity, reaction time and fluid IQ. Sensory and cognitive performance was reassessed after ~4 years (N = 2,438 to 17,659). In statistical modelling including age, sex, socioeconomic status, educational level, smoking, maternal smoking and comorbid disease, adult height was positively associated with sensory and cognitive function (partial correlations; *pr* 0.05 to 0.12, *p* < 0.001). Within the normal range of birth weight (10^th^ to 90^th^ percentile), there was a positive association between birth weight and sensory and cognitive function (*pr* 0.06 to 0.14, *p* < 0.001). Neither adult height nor birth weight was associated with change in sensory or cognitive function. These results suggest that adverse prenatal and childhood experiences are a risk for poorer sensory and cognitive function and earlier development of sensory and cognitive impairment in adulthood. This finding could have significant implications for preventing sensory and cognitive impairment in older age.

## Introduction

Prenatal and early childhood development have a critical effect on long-term health in adulthood [1]. Early development may affect adult susceptibility to a range of non-communicable disease including cardiovascular disease [2] and diabetes [3]. The aim of this study was to examine the associations between prenatal and early childhood development with cognitive, vision and hearing function in middle age. Commonly used indexes of prenatal development include birth weight and other measures of body size at birth. Measures of body size at birth represent an indirect, summative measure of influences on the developing foetus [4]. In contrast to measures of body size at birth (an index of prenatal exposure), measures of adult leg length and height are sensitive to environmental factors and nutrition in early childhood that impact on growth [5].

Birth weight and post-natal growth during early childhood and adolescence are positively associated with cognitive ability in childhood [6–17]. There is less research on whether early life development affects cognitive performance in adulthood and old age, and the findings in the existing literature are mixed. Some studies of middle aged and older adults did not detect reliable associations between early life development and cognitive function in adulthood and older age [18–20]. One possible explanation may be that early life development exerts a stronger influence on cognition in childhood than later in life. Richards and colleagues [16] assessed cognitive function in 3,900 participants at ages 8, 11, 15, 26 and 43 years. Birth weight was positively associated with cognitive ability at 8, 11, 15 and 26 years, though there was no association at 43 years. Other studies do support associations between both prenatal [21,22] and childhood [22,23] development and adult cognitive function. In summary, there is inconsistent evidence that pre-natal growth may affect cognitive ability in adulthood. Previous research findings are also inconsistent in relation to early life development and age-related *change* in cognitive function. Martyn et al [19] and Shenkin et al [20] reported no association between any anthropometric parameter at birth and estimated cognitive decline. Gale and colleagues [24] found associations between cognitive decline and adult head circumference, but not head circumference at birth.

To date, most research attention has focused on the long-term cognitive outcomes of low birth weight babies and birth weight within the normal range. However, there is also evidence that unusually large babies may have poorer cognitive outcomes. Large babies are defined as being outside the normal range of distribution according to weight, head circumference or length (i.e. greater than the 90^th^ percentile or 97^th^ percentile, depending on the cut-point used [25]). Maternal diabetes and obesity are associated with higher likelihood of a large baby [26]. Cesur and Rashad [27] analysed two longitudinal data sets, examining patterns of academic performance in junior school for a combined sample of 19,280 children. Children who had either low or high birth weight had poorer academic performance compared to children within the normal range of birth weight. Given increasing rates of diabetes and obesity globally [28,29] and parallel evidence of increasing numbers of babies who are large for gestational age [30], adverse developmental outcomes associated with overweight babies is likely to assume increased importance.

Few studies have investigated associations between early life development and hearing and visual sensory function. Birth weight was reported to be associated with likelihood of having state registration for hearing impairment [31], hearing and visual acuity [32] and self-reported hearing problems [33]. Sayer et al [34] reported that reduced growth in childhood (but not birth weight) was associated with lens opacity (but not visual acuity) and poorer hearing levels when assessed around age 67 years. Barrenas [35] reported that adult height was associated with hearing, and concluded that adverse early life experience may convey increased susceptibility to hearing loss in adulthood. However, not all studies support associations between prenatal and/or childhood development and adult sensory function; Ecob and colleagues [36] reported no association between birth weight or BMI during childhood and hearing assessed at age 45 years.

It may be that the association between indices of early life development and adult cognition and sensory function is due to confounding; factors including socio-economic status or birth trauma may account for the association, despite attempts to control statistically for potential confounds. However, there is evidence from experimental studies with animal models that supports observational findings in humans for the influence of early life exposures on development of cognitive and sensory systems [37]. It is plausible that there is a direct causal relationship between early development and neurosensory function in adulthood, although the mechanism is unclear. It may be that under-nutrition impacts on development of the brain and sensory organs [38], or there may be alterations in gene expression that affect cognitive and sensory function [39–41]. Alternatively, glucocorticoid hormones or growth factors may be modulated by early experience and impact on neurosensory development [35,42–44]. It is also possible that the effect of early development on cognitive and sensory function is via increased susceptibility to diabetes and cardiovascular disease; adverse early development is associated with increased risk of cardiovascular disease and diabetes [2,3], and cognitive decline and poor hearing and visual function are independently associated with cardiovascular disease and diabetes [45–48].

It is unclear what the relative contribution of prenatal versus childhood development is for adult cognitive and sensory function and decline in function with age. In the present study, we examined hearing, vision and cognitive function in middle age according to birth weight (an index of prenatal development) and adult height (an index of early childhood development) in a very large sample of middle aged people, thereby increasing power for detecting small effects later in life. We also examined change in hearing, vision and cognitive function longitudinally over ~4 years as a function of birth weight and adult height. We hypothesized that i) cognitive and sensory function would be associated with birth weight, with poorer performance for low and high birth weight and better performance for larger babies within the normal range; ii) taller adults would have better cognitive and sensory function than shorter adults; iii) low and high birth weights and short adult height would be associated with a greater decline in sensory and cognitive function.

## Methods

### The UK Biobank sample

This study utilised data from the UK Biobank resource [49]. UK Biobank is an open access resource that is open to bona fide scientists undertaking health-related research that is in the public good. Approved scientists from the UK and overseas and from academia, government, charity and commercial companies can use the Resource (for details of access, see http://www.ukbiobank.ac.uk/scientists-3/). Ethical approval for UK Biobank was obtained from the National Health Service North West Multi-centre Research Ethics Committee. During 2006–2010, 503,325 participants were recruited, with a response rate of 5.47%. As data collection proceeded, additional measures were added to the test protocol. Thus, different numbers of participants completed each measure (Table 1). For the analysis of visual acuity, participants were excluded if they required glasses for distance viewing, but had completed visual acuity testing without them. Participants attended a UK Biobank assessment centre and provided informed consent. Participants completed a 90 minute assessment that included i) a computerised questionnaire on lifestyle, environment and medical history, ii) physical measures including hearing and vision testing. A description of the procedure and additional data collected may be found elsewhere (http://www.ukbiobank.ac.uk/). Data on sex, ethnicity and area of residence were collected for each participant. Areas of residence were translated to Townsend deprivation scores to provide a proxy measure of socioeconomic status. Townsend scores are widely used in health studies, and are applicable across the countries of the UK [50]. Townsend scores for small geographical areas (Electoral wards in England, Wales and Northern Ireland, postal sectors in Scotland) are based on four variables including employment, car ownership, home ownership and household occupancy. Each variable is translated to a z-score relative to the national level. Z-scores are then summed to give a single deprivation score for each area. Lower Townsend scores represent areas associated with more affluent socioeconomic status.

**Table 1:** Demographic characteristics of participants in the UK Biobank study subsamples for sex, ethnicity and socio-economic status (Townsend deprivation index).

	Hearing		Vision		IQ		Reaction time	
	Baseline	Repeat assessment	Baseline	Repeat assessment	Baseline	Repeat assessment	Baseline	Repeat assessment
**Birth weight** subsample size (n)	80,572	2,551	37,505	1,454	81,083	2,630	241,660	9,579
Sex (Male)	39.0%	42.0%	38.6%	40.8%	38.9%	42.0%	38.9%	41.3%
Ethnicity (White)	95.2%	97.9%	94.4%	97.7%	95.4%	97.9%	96.8%	98.4%
Mean Townsend score* (SD)	-1.3 (2.8)	-2.1 (2.5)	-1.2 (2.8)	-2.2 (2.5)	-1.3 (2.8)	-2.1 (2.5)	-1.4 (3.0)	-2.0 (2.6)
**Adult height** subsample size (n)	144,404	4,425	65,705	2,438	144,362	4,546	433,930	17,659
Sex (Male)	45.5%	49.2%	45.3%	48.2%	45.4%	49.4%	45.5%	48.5%
Ethnicity (White)	91.6%	96.6%	90.5%	96.6%	92.2%	96.8%	94.6%	97.7%
Mean Townsend score* (SD)	-1.1 (2.9)	-2.0 (2.6)	-1.1 (2.9)	-2.2 (2.5)	-1.2 (2.9)	-2.0 (2.6)	-1.3 (3.1)	-2.0 (2.7)

* Lower Townsend scores represent areas associated with more affluent socioeconomic status.

During 2012 and 2013, 17,819 participants were recruited for repeat assessment, with a response rate of 21%. All baseline and physical measures were repeated, including hearing, vision and cognition. The average interval between baseline testing and repeat assessment was 4 years, and ranged between 2 and 7 years. (For further details of the repeat assessment, see http://biobank.ctsu.ox.ac.uk/~bbdatan/Repeat_assessment_doc_v1.0.pdf).

### Birth weight and adult height

Self-reported birth weight was recorded for those participants who reported that they knew their own birth weight. Self-reported birth weight is a valid measure of birth weight [51,52]. Participants’ standing height was measured in centimeters using a Seca 202 telescopic measuring rod (http://www.seca.com/).

### Hearing

Hearing was assessed with the Digit Triplet Test (DTT[53]), a speech-in-noise test that is used internationally for large-scale hearing screening. A detailed description of the DTT is provided elsewhere (http://biobank.ctsu.ox.ac.uk/crystal/label.cgi?id=100049). Briefly, fifteen sets of three monosyllabic digits were presented over circumaural headphones (Sennheiser HD-25). Participants first set the volume of the stimuli to a comfortable level. Each ear was tested separately with the order of testing randomised across participants. Digits were presented in a background of noise shaped to match the spectrum of the speech stimuli. Noise levels varied adaptively, contingent on recognition of the three digits, to estimate the signal-to-noise ratio (SNR) for a criterion performance of 50% correct. The speech recognition threshold was calculated from the mean SNR for the last eight triplets. Lower (more negative) scores represent better performance. The recognition threshold for the better ear was used as the index of hearing.

### Vision

Visual acuity testing was based on reading high contrast letters with the participant seated at a distance of 4 metres. Visual acuity measures were completed on both eyes. Participants wore glasses or contact lenses that were normally worn for distance viewing. Visual acuity scores were determined as the logMAR size at which 3 out of the 5 letters presented were read correctly. A detailed description of visual acuity testing is provided elsewhere (http://biobank.ctsu.ox.ac.uk/crystal/refer.cgi?id=100250). Visual acuity for the better eye was used as the index of vision.

### Cognitive tests

Cognitive tests were completed via a computerised touchscreen interface. A detailed description of cognitive testing is reported elsewhere (http://biobank.ctsu.ox.ac.uk/crystal/label.cgi?id=100026). Processing speed testing was based on the card game ‘Snap’. Participants were shown two pairs of cards out of a set of 12 pairs. If both cards displayed a matching symbol, participants pressed a response button as quickly as possible using their dominant hand. Reaction time was taken as the average time to correctly respond to a matching pair. Fluid intelligence (IQ) (the capacity for logical thought and problem solving, independent of acquired knowledge) was estimated via multiple choice responses to questions such as "Bud is to Flower as Child is to?" Participants had 2 minutes to complete as many questions as possible out of 13. Correct responses scored 1 point, while questions that were answered incorrectly or that were not completed within the time limit scored zero.

### Cardiovascular disease, hypertension, cholesterol and diabetes

Cardiovascular disease was identified on the basis of participant report of any cardiovascular problems, including heart attack, stroke, heart failure, angina, transient ischemic attack, intermittent claudication, arterial embolism or deep venous thrombosis. Hypertension was identified if the participant reported that they had high blood pressure, currently took medication for high blood pressure, or had a measured systolic blood pressure greater than 140 mm Hg or diastolic pressure greater than 90 mm Hg. High cholesterol was identified if the participant reported that they had high cholesterol, or that they were currently taking medication for high cholesterol. Diabetes was identified if the participant reported that they had Type 1 or Type 2 diabetes, or that they took insulin for diabetes.

### Education level, smoking and maternal smoking

Education level was categorised according to the highest self-reported level of study (University or college degree or other professional qualification; A/AS level, O level, GCSE or vocational qualification; no qualification post primary school). Maternal smoking was recorded in response to the question "Did your mother smoke regularly around the time when you were born?" Smoking status was based on self-report of previous or current tobacco smoking (current/previous/never).

### Data analysis

Participants were fractionally ranked by birth weight and adult height separately for males and females. Fractional ranks were then pooled across sexes to provide percentile ranks by birth weight and by height within the sample independent of sex. Plots of reaction time, IQ, hearing and vision acuity by percentile rank for birth weight and adult height were generated. To index change in performance over time, performance at the baseline assessment was subtracted from performance on repeat assessment. Change in performance was then divided by the time between baseline and repeat assessment to provide a figure for the average annual change in performance for the hearing, vision and cognitive tests.

To analyse the association between adult height and hearing, vision and cognition, a linear regression model was applied with cognitive or sensory performance as the dependent variable, adult height as the independent variable, with age, sex, Townsend deprivation index quintile, educational level, smoking, diabetes, cardiovascular disease, hypertension, high cholesterol and maternal smoking as possible confounds. Similar analyses were carried out to analyse the association between adult height and change in hearing, vision and cognition.

To analyse the association between birth weight and hearing, vision and cognition, linear regression models were applied for those participants within the normal range (10^th^ to 90^th^ percentile) of birth weight with age, sex, Townsend deprivation index quintile, educational level, smoking, diabetes, cardiovascular disease, hypertension, high cholesterol and maternal smoking as possible confounds. Similar analyses were carried out to analyse the association between birth weight and change in hearing, vision and cognition. To compare the smallest and largest extremes of the distribution by birth weight, the top and bottom 3% were compared to the 3% of the sample in the middle of the distribution (i.e. +/- 1.5% of the sample either side of the 50 percentile). An ANOVA model was applied, hearing, vision and cognition as the dependent variable and group (bottom, middle or top 3% of the distribution) as the independent variable in the model, with the covariates age, sex, Townsend deprivation index quintile, educational level, smoking, diabetes, cardiovascular disease, hypertension, high cholesterol and maternal smoking. All analyses were performed with SPSS version 20.0 (http://www-01.ibm.com/software/analytics/spss/).

## Results

Table 1 shows the number and demographic characteristics of the subsets of participants who completed the hearing, vision and cognitive tests and for whom birth weight or adult height data were available. Table 2 describes the average birth weight and height for males and females in each birth weight and height percentile.

**Table 2:** Average birth weight and height for sample population percentiles for males and females.

	Males		Females	
Percentile	Birth weight (kg)	Height (cm)	Birth weight (kg)	Height (cm)
<3	1.69	159	1.62	147
3 to 10	2.54	165	2.31	152
10 to 20	2.89	168	2.71	156
20 to 30	3.14	171	2.91	158
30 to 40	3.25	173	3.05	160
40 to 50	3.37	174	3.19	161
50 to 60	3.50	176	3.32	163
60 to 70	3.64	178	3.42	165
70 to 80	3.81	180	3.36	166
80 to 90	4.10	182	3.84	168
90 to 97	4.49	186	4.18	172
>97	5.14	191	4.72	177

### Cognitive and sensory function in adulthood

#### Adult height

**Fig 1:**
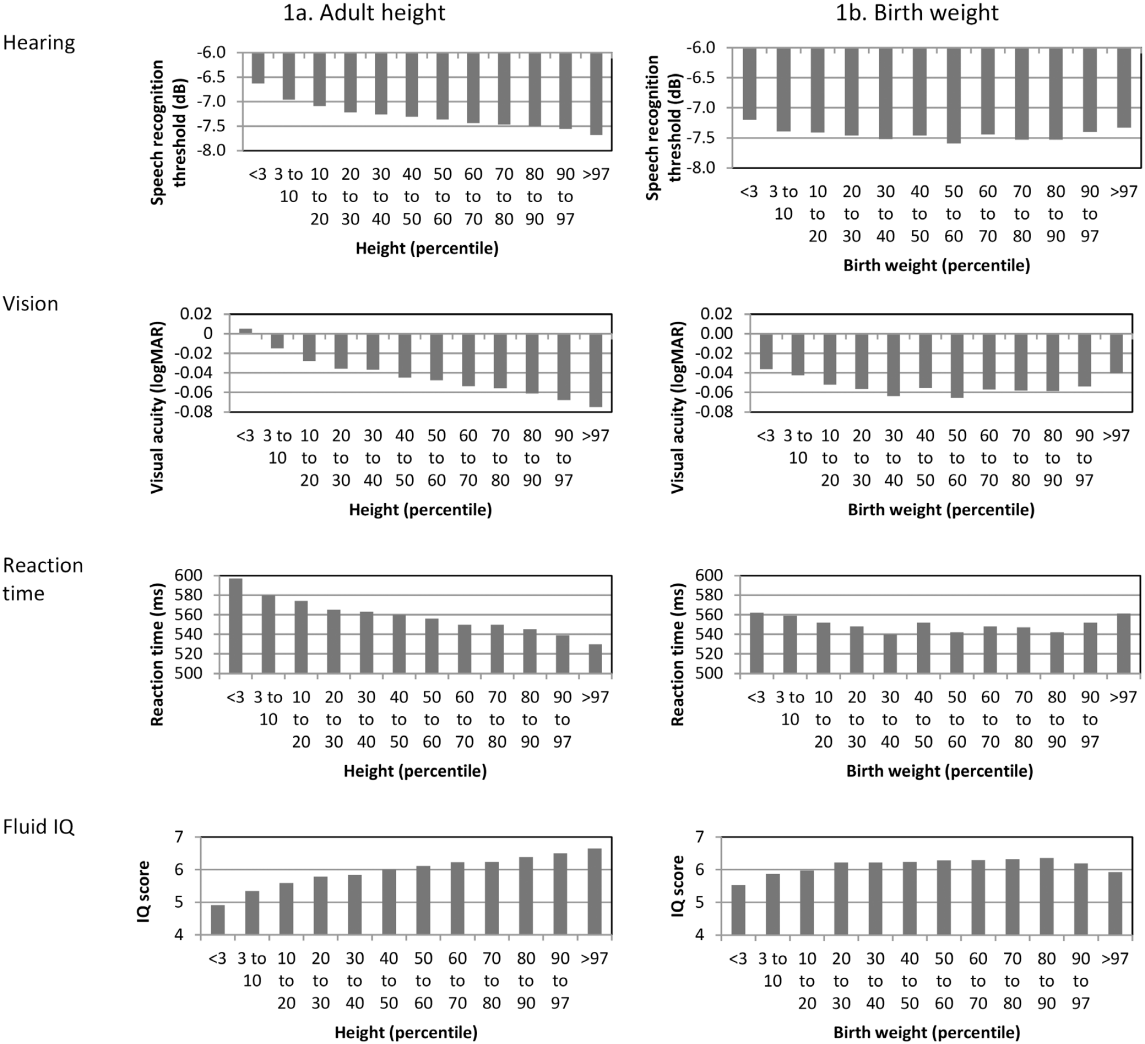
Hearing, vision, reaction time and fluid IQ in adulthood by adult height and birth weight percentile.

Fig 1 (left hand side, ‘1a. Adult height’) shows hearing, vision and cognitive performance by percentiles of adult height. Regression models including height, sex, age, Townsend quintile, smoking status, cardiovascular disease, diabetes, hypertension, high cholesterol and maternal smoking and hearing, vision, reaction time and IQ were all statistically significant (Hearing: *r* ^2^ = 0.06, F(11,120783) = 756, *p* < 0.001; Vision: *r* ^2^ = 0.08, F(11,54922) = 402, *p* < 0.001; Reaction time: *r* ^2^ = 0.10, F(11,358036) = 3642, *p* < 0.001; IQ: *r* ^2^ = 0.16, F(11,120857) = 2073, *p* < 0.001). Adult height was a significant contributor to each regression model (Table 3, left hand side ‘3a. Function in adulthood’). Partial correlations (*pr*) for height controlling for sex, age, Townsend quintile, smoking status, cardiovascular disease, diabetes, hypertension, high cholesterol and maternal smoking were: hearing 0.06; vision 0.06, reaction time 0.05; IQ 0.12.

**Table 3:** Statistics for regression models for adult height and birth weight and hearing, vision and cognitive function in adulthood and 4-year change in function.

	3a. Function in adulthood		3b. 4-year change in function	
	Height	Birth weight	Height	Birth weight
**Hearing**	β	β	β	β
Height / Birth weight	-0.06***	-0.01**	-0.02	0.02
Sex	0.00	-0.02***	0.02	0.01
Age	0.18***	0.22***	0.04*	0.05*
Townsend Quintile	0.09***	0.06***	0.03	0.07**
Education level	0.06***	0.05***	0.04*	0.04
Smoking	0.01**	-0.01	0.01	0.01
Cardiovascular Disease	0.02***	0.02***	0.01	0.00
Diabetes	0.04***	0.02***	0.03	-0.04
Hypertension	0.00	0.00	0.03	0.02
Cholesterol	0.02***	0.02***	-0.01	0.01
Maternal smoking	-0.05***	-0.03***	0.02	0.03
**Vision**				
Height / Birth weight	-0.06***	-0.01*	-0.03	0.02
Sex	-0.07***	-0.08***	0.01	0.03
Age	0.21***	0.23***	0.04	0.04
Townsend Quintile	0.07***	0.06***	0.00	0.01
Education level	0.07***	0.07***	0.03	0.05
Smoking	-0.01	-0.01	0.02	0.01
Cardiovascular Disease	0.01**	0.01	-0.03	-0.05
Diabetes	0.03***	0.03***	0.01	0.04
Hypertension	0.01**	0.01*	0.00	0.00
Cholesterol	-0.01	-0.01	-0.03	-0.04
Maternal smoking	-0.01***	0.00	0.01	0.04
**Reaction time**				
Height / Birth weight	-0.05***	-0.01***	-0.01	0.01
Sex	-0.08***	-0.09***	0.01	-0.01
Age	0.26***	0.29***	-0.06***	-0.06***
Townsend Quintile	0.07***	0.06***	0.00	0.01
Education level	0.06***	0.06***	0.00	-0.01
Smoking	0.01***	0.00	0.01	0.02
Cardiovascular Disease	0.02***	0.01***	-0.02	-0.02
Diabetes	0.03***	0.02***	0.00	0.02
Hypertension	0.00	0.00	0.00	0.00
Cholesterol	0.01***	0.01***	0.03*	0.02
Maternal smoking	-0.04***	-0.03***	-0.01	0.00
**Fluid IQ**				
Height / Birth weight	0.12***	0.03***	-0.01	-0.03
Sex	0.06***	0.07***	0.00	-0.01
Age	0.02***	0.00	-0.02	0.02
Townsend Quintile	-0.09***	-0.06***	0.06***	0.07**
Education level	-0.34***	-0.35***	0.14***	0.15***
Smoking	-0.01*	0.01	0.02	0.03
Cardiovascular Disease	-0.02***	-0.02***	0.02	0.03
Diabetes	-0.03***	-0.02***	0.02	0.00
Hypertension	-0.02***	-0.02***	0.00	0.01
Cholesterol	-0.01***	-0.01*	0.01	0.01
Maternal smoking	0.03***	0.01**	-0.01	-0.02

β is the standardised coefficient from the multiple regression model.

***p < 0.001,

**p < 0.01,

*p < 0.05.

#### Birth weight

Fig 1 (right hand side, ‘1b. Birth weight’) shows hearing, vision and cognitive performance by birth weight percentile. Within the normal range of birth weight (10^th^ to 90^th^ percentile), regression models including birth weight, sex, age, Townsend quintile, smoking status, cardiovascular disease, diabetes, hypertension, hypercholesterolemia and maternal smoking and hearing, vision, reaction time and IQ were all statistically significant (Hearing: *r* ^2^ = 0.06, F(11,55158) = 781, *p* < 0.001; Vision: *r* ^2^ = 0.07, F(11,25737) = 187, *p* < 0.001; Reaction time: *r* ^2^ = 0.11, F(11,163368) = 1808, *p* < 0.001; IQ: *r* ^2^ = 0.14, F(11,55586) = 837, *p* < 0.001). Birth weight made a statistically significant contribution to each model (Table 3, left hand side ‘3a. Function in adulthood’). Partial correlations (*pr*) for birth weight controlling for sex, age, Townsend quintile, smoking status, cardiovascular disease, diabetes, hypertension, cholesterol and maternal smoking were: hearing 0.04; vision 0.01, reaction time 0.01; IQ 0.12.

The top and bottom 3% by birth weight were compared with the middle 3% (centred on the 50^th^ percentile). For an ANOVA including birth weight category (low, middle, high) and potential confounds there were significant differences in hearing, vision, reaction time and IQ across birth weight category (Table 4). Bonferroni post-hoc comparisons revealed that for hearing, vision, reaction time and IQ, the middle category had significantly better performance than both the low and high categories (both *p* < 0.001). The high category had significantly better performance than the low category for hearing and IQ (*p* = 0.01; *p* < 0.001). There was no difference between the low and high categories in reaction time or vision (*p* = 0.93; *p* = 0.43).

**Table 4:** Statistics for ANOVA models for birth weight category and hearing, vision, reaction time and fluid IQ in adulthood.

	Hearing		Vision		Reaction time		Fluid IQ	
	df	F	df	F	df	F	df	F
Birth weight category (low, middle, high)	2, 7269	7.77***	2, 4874	5.78**	2, 2304	5.16**	2, 7289	45.76***
Age	1, 7269	235.23***	1, 4874	178.95***	1, 2304	119.01***	1, 7289	1.31
Sex	1, 7269	0.60	1, 4874	31.89***	1, 2304	18.49***	1, 7289	47.49***
Townsend Quintile	3, 7269	15.49***	3, 4874	9.16***	3, 2304	4.24**	3, 7289	13.41***
Education level	3, 7269	17.13***	3, 4874	5.02**	3, 2304	5.20**	3, 7289	358.94***
Smoking	2, 7269	0.46*	2, 4874	5.74**	2, 2304	3.14*	2, 7289	3.31*
Diabetes	1, 7269	4.42	1, 4874	1.32	1, 2304	0.79	1, 7289	0.05
Cardiovascular disease	1, 7269	3.90*	1, 4874	0.00	1, 2304	0.31	1, 7289	4.90*
Cholesterol	1, 7269	0.35	1, 4874	5.55*	1, 2304	3.88*	1, 7289	2.02
Hypertension	1, 7269	0.01	1, 4874	0.04	1, 2304	0.11	1, 7289	0.15
Maternal smoking	1, 7269	2.22	1, 4874	0.21	1, 2304	0.09	1, 7289	0.24

***p < 0.001,

**p < 0.01,

*p < 0.05.

### Change in sensory and cognitive function

#### Adult height

**Fig 2:**
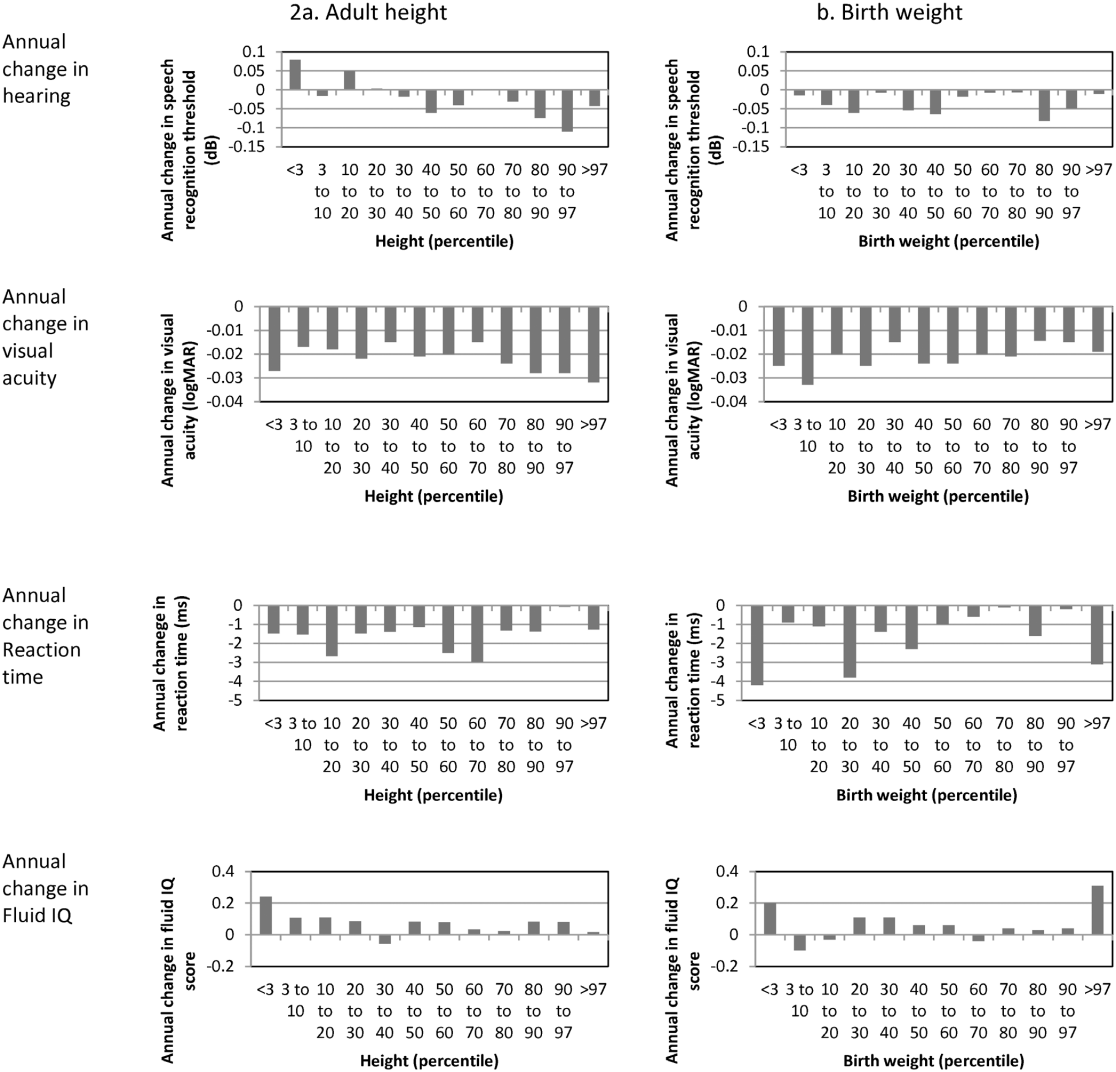
Change in hearing, vision, reaction time and fluid IQ by adult height and birth weight percentile. Negative scores correspond to worse performance over time.

Fig 2 (left hand side, ‘2a. Adult height’) shows the annual change in hearing, vision, reaction time and IQ by percentile of adult height. For all figures, positive scores correspond to improvement in performance while negative scores correspond to worse performance over time. For vision and reaction time, there was a uniform pattern of worse performance over time. For hearing, the lowest height group showed a tendency for improved performance. For IQ, there was a tendency for improved performance across almost all percentile groups.

Regression models (Table 3, right hand side ‘3b. 4-year change in function’) including height, sex, age, Townsend quintile, smoking status, cardiovascular disease, diabetes, hypertension, high cholesterol and maternal smoking and change in hearing, vision, reaction time and IQ were statistically significant for change in hearing, reaction time and IQ (Hearing: *r* ^2^ = 0.01, F(11,3768) = 3.43, *p* < 0.001; Reaction time: *r* ^2^ = 0.004, F(11,11372) = 4.20, *p* < 0.001; Fluid IQ: *r* ^2^ = 0.03, F(11,3875) = 9.5, *p* < 0.001). The regression model for change in visual acuity was not statistically significant (*r* ^2^ = 0.01, F(11,2079) = 0.99, *p* = 0.45). Height was not a significant contributor to any model. For hearing, older age and lower educational attainment was associated with improved function. Older age and high cholesterol was associated with slower reaction time at reassessment. More deprived Townsend quintile and lower educational attainment was associated with improved IQ over time.

#### Birth weight

Fig 2 (right hand side, ‘2b. Birth weight’) shows the annual change in hearing, vision, reaction time and IQ by percentile of birth weight. Patterns of change were similar to those for adult height. There was a tendency for worse performance over time for hearing, vision and reaction time across the range of birth weight. Small improvements were evident for fluid IQ. Within the normal range of birth weight (10^th^ to 90^th^ percentile), regression models including birth weight, sex, age, Townsend quintile, smoking status, cardiovascular disease, diabetes, hypertension, cholesterol and maternal smoking were statistically significant for change in hearing, reaction time and IQ (Hearing: *r* ^2^ = 0.013, F(11,1767) = 2.17, *p* = 0.01; Reaction time: *r* ^2^ = 0.076, F(11,5004) = 2.62, *p* = 0.002; IQ: *r* ^2^ = 0.032, F(11,1825) = 5.42, *p* < 0.001). The model for change in vision was not statistically significant (*r* ^2^ = 0.01, F(1,1027) = 0.89, *p* = 0.55). Birth weight was not a significant contributor to any model (Table 3, right hand side ‘3b. 4-year change in function’). The top and bottom 3% by birth weight were compared with the middle 3% (centred on the 50^th^ percentile). In ANOVA including birth weight category (low, middle, high) and potential confounders, there were no significant differences in birth weight category for change in performance (Table 5).

**Table 5:** Statistics for ANOVA models for birth weight category and 4-year change in hearing, vision, reaction time and fluid IQ.

	Hearing		Vision		Reaction time		Fluid IQ	
	df	F	df	F	df	F	df	F
Birth weight category (low, middle, high)	2, 213	0.93	2, 113	0.57	2, 687	0.09	2, 221	2.95
Age	1, 213	1.01	1, 113	0.06	1, 687	5.81*	1, 221	1.47
Sex	1, 213	0.15	1, 113	0.04	1, 687	0.13	1, 221	0.27
Townsend Quintile	3, 213	0.09	3, 113	1.25	3, 687	0.38	3, 221	0.60
Education level	3, 213	1.80	3, 113	1.51	3, 687	2.35	3, 221	1.34
Smoking	2, 213	4.19*	2, 113	2.98	2, 687	0.21	2, 221	1.30
Diabetes	1, 213	0.00	1, 113	1.67	1, 687	0.13	1, 221	2.55
Cardiovascular disease	1, 213	0.01	1, 113	0.27	1, 687	1.53	1, 221	0.04
Cholesterol	1, 213	1.50	1, 113	0.45	1, 687	0.00	1, 221	0.48
Hypertension	1, 213	0.06	1, 113	1.83	1, 687	0.81	1, 221	1.02
Maternal smoking	1, 213	0.34	1, 113	0.00	1, 687	0.01	1, 221	1.46

*p < 0.05.

## Discussion

In statistical models that included age, sex, socioeconomic status, educational level, diabetes, cardiovascular disease, hypertension, high cholesterol, smoking status and maternal smoking, adult height was linearly related to hearing, vision and cognitive function in middle age, with taller adults having better function. Environmental factors and nutrition in early childhood (indexed by adult height) are thus related to sensory and cognitive function in middle age. Birth weight showed a different pattern of association. Within the normal range (10^th^ to 90^th^ percentile), larger birth weight was associated with better hearing, vision and cognitive function. Participants with both the smallest and the largest birth weights had significantly poorer function than those within the normal range. Adult cognitive and sensory function are therefore related to prenatal experience (indexed by birth weight). It is striking that effects of early development persist into middle age, after decades of life experiences that impact on cognitive and sensory functioning. Early life factors may also interact with environmental exposures during the lifespan to increase susceptibility to cognitive and sensory impairment [54]. Common influence by early life factors may explain the observation that cognitive and sensory performance are associated, independently of age [55].

On reassessment after ~4 years, improvements in the IQ and the hearing measure were apparent. Performance on the vision and reaction time tasks showed a more expected pattern of decline in performance. Practice effects are well-known in studies of cognitive aging and may affect some tests more than others [56], and it is problematic to dis-entangle practice effects from age-related changes in performance. Neither adult height nor birth weight were consistently associated with change in sensory or cognitive function over time. Prenatal and early childhood development were associated with the level of sensory and cognitive functioning in adulthood, but not to the rate of change in function with age, as indexed by the measures used in the present study. Note that adverse early life experience may still be a risk for earlier development of sensory and cognitive impairment. Although the rate of change with age may be similar, those who begin from a lower level of performance will reach the threshold of impairment sooner (cf the concept of ‘cognitive reserve’ and brain ageing [57]). This finding may have significant implications for preventing sensory and cognitive impairment in older age; modelling suggests that even relatively modest delays in cognitive decline would substantially reduce the number of cases of dementia in the population [58]. The same may be true for hearing and vision impairment. Consistent with previous research, indices of early life development were associated with a small amount of variance in cognitive and sensory performance. For example, across the range of adult height, the effect on hearing corresponded to a 1 dB difference in signal to noise ratio for speech recognition (the mean difference in signal to noise ratio for speech recognition between the ages of 40 and 69 years is 1.2 dB [59]), a 0.08 logMAR difference in visual acuity, a difference of 67 ms in reaction time and 1.7 points on a 13-point scale of fluid IQ. Even if modest in size, the effects of prenatal and childhood development are universal and may thus be significant determinants of cognitive and sensory aging at a population level [37]. Shifts in the mean level of function within a population have dramatic effects on the numbers of people that fall within the range of clinical impairment. For example, a decrease in the mean IQ of the population by 5 points on a standard distribution would double the number of people with an IQ <70 [37]. The effect of early development on cognitive aging has been recognised as a research priority [60,61]. Effects of early development in relation to vision and hearing impairment in adulthood and old age may warrant similar attention.

The present study has some limitations. The study was observational with cross-sectional and longitudinal measurement, and it is not possible to establish a causal link between early development factors and adult cognitive and sensory function. We attempted to control statistically for potential confounds such as socioeconomic status, educational level, age, comorbid disease, smoking and maternal smoking. However, it is possible that unmeasured confounds or insufficient control of measured confounds could impact results. For example, no information was available on gestational age or parental body size. Ideally, body size measures at birth should be corrected for parental size and gestational age in order to distinguish between growth appropriate for gestational age and growth restriction [62], though the necessary data are often unavailable. Even with correction for gestational age and parental size, body size at birth does not allow insight into the aetiology of low birth weight, for example whether due to acute injury resulting in impaired growth or slow growth from conception [63]. The UK Biobank sample had a low response rate (5.47%) and includes a higher proportion of white, female and less deprived people than in the general UK population. However, due to the very large and inclusive nature of the sample, associations between risk factors and health outcomes can be made with confidence [49]. Different numbers of participants completed the different tests and participated in the reassessment. In particular, reassessment included lower numbers of participants than the baseline assessment, so the analysis of change in cognitive and sensory function is statistically lower-powered than the cross-sectional analysis. However, sample sizes for reassessment remained sufficiently large (2,438 to 17,659, depending on the measures) and compare favourably with previously published studies with much smaller samples.

Sensory and cognitive impairments in older age are a substantial and increasing source of poor quality of life and economic costs globally [64]. As with other diseases that are linked to early development, cognitive and sensory function appears to be influenced by the full range of development; not just those within the extremely deprived range, but also those within the normal range. In models of infectious disease, individuals remain healthy until they contract the disease. In contrast, non-communicable diseases like diabetes, cardiovascular disease, cognitive impairment and sensory impairment develop gradually over the life course. Early development may programme a metabolic trajectory for the lifespan which interacts with accumulated challenges to health (such as obesity, smoking or hypertension [35]) and age-related declines in physiological plasticity [65]. One implication of this model is that the effectiveness of interventions in adulthood are relatively limited, while intervention in a developmentally more plastic period is likely to have a much larger impact in altering the metabolic trajectory and preventing disease [65]. In order to reduce the burden of cognitive decline and sensory impairment, future research attention may centre on identifying avenues for intervention to optimise foetal growth and childhood development [62]. Public health policy should also take into account the importance of focusing on interventions during early life, with potentially greater effect than attempting to mitigate cognitive decline and sensory impairment in adulthood and older age.
